# Intravenous Administration of Scutellarin Nanoparticles Augments the Protective Effect against Cerebral Ischemia–Reperfusion Injury in Rats

**DOI:** 10.1021/acs.molpharmaceut.1c00942

**Published:** 2022-04-20

**Authors:** Chang Yang, Qing Zhao, Shanshan Yang, Libin Wang, Xingyuan Xu, Lisu Li, Wafa T. Al-Jamal

**Affiliations:** † State Key Laboratory of Functions and Applications of Medicinal Plants/ Guizhou Provincial Key Laboratory of Pharmaceutics, Guizhou Medical University, Guiyang, Guizhou 550004, China; ‡ Engineering Research Center for the Development and Application of Ethnic Medicine and TCM (Ministry of Education), Guizhou Medical University, Guiyang, Guizhou 550004, China; § School of Pharmacy, Queen’s University Belfast, Belfast BT9 7BL, United Kingdom

**Keywords:** scutellarin, PLGA nanoparticles, stroke, cerebral ischemia, traditional Chinese medicine (TCM)

## Abstract

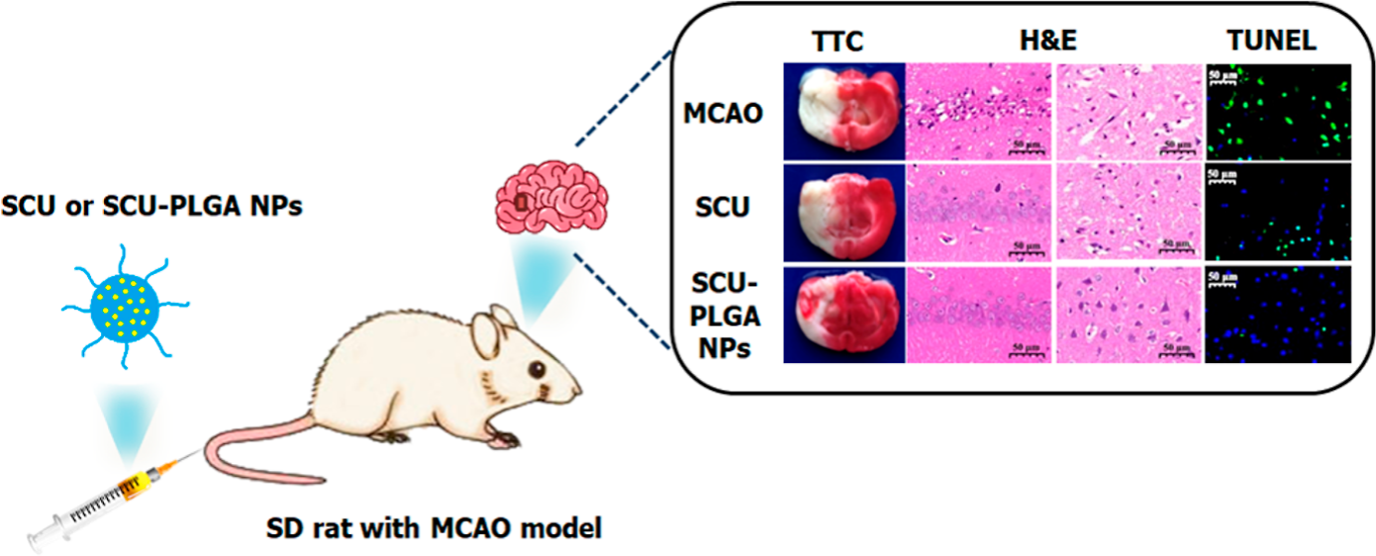

This study investigates the protective effect of poly(lactic-*co*-glycolic acid) (PLGA) nanoparticles (NPs) loaded with scutellarin (SCU), a flavone isolated from the traditional Chinese medicine*Erigeron breviscapus* (Vant.) Hand.-Mazz., in reducing cerebral ischemia/reperfusion (I/R) injury *in vivo*. The focal cerebral I/R injury model was established by occluding the middle cerebral artery for 1 h in male Sprague-Dawley (SD) rats. Our SCU-PLGA NPs exhibited an extended *in vitro* release profile and prolonged blood circulation in rats with cerebral ischemia. More importantly, when administered intravenously once a day for 3 days, SCU-PLGA NPs increased the SCU level in the ischemic brain, compared to free SCU, resulting in a significant reduction of the cerebral infarct volume after cerebral I/R. Furthermore, SCU-PLGA NPs reversed the histopathological changes caused by cerebral I/R injury, as well as attenuated cell apoptosis in the brain tissue, as confirmed by hematoxylin and eosin, and TUNEL staining. Our findings have revealed that our injectable SCU-PLGA NPs provide promising protective effects against cerebral I/R injury, which could be used in combination with the existing conventional thrombolytic therapies to improve stroke management.

## 1 Introduction

Stroke is the second leading cause of death and a major cause of disability worldwide.^1^ Its incidence is increasing due to the aging population. According to the Report on Cardiovascular Diseases in China 2018, 13 million of the approximately 290 million patients with cardiovascular disease have strokes. On an average, one in five adults suffers from cardiovascular and cerebrovascular diseases. Based on the limited efficacy obtained so far using Western medications, clinicians have begun exploring traditional Chinese medicine (TCM) in stroke prevention and treatment.

Breviscapine is a flavonoid mixture extracted from the Chinese herb*Erigeron breviscapus* (Vant.) Hand.-Mazz, with its main active constituent (>90%) being scutellarin (SCU). Several studies have reported the beneficial effects of breviscapine against the cerebrovascular diseases, including protection against ischemia/reperfusion (I/R), anti-inflammation, anticoagulation, and antithrombosis.^2−4^ Breviscapine has been widely used to treat coronary heart diseases, such as angina, myocardial ischemia, cerebral ischemia, and cerebral thrombosis.^2−4^ Approved injections, tablets, and granules of breviscapine are available in China.^2^ Several breviscapine nanoformulations, such as lipid emulsion,^5−7^ solid lipid nanoparticles (NPs),^8^ mesoporous silica NPs,^9^ nanosuspensions,^10^ and poly(lactic-*co*-glycolic acid) (PLGA) microparticles,^11^ were developed; however, they have failed to produce therapeutic outcomes that are comparable to SCU, even at much higher doses, probably due to the presence of other flavonoids that counteract the SCU activity.^12^

SCU, the main active component of breviscapine, has poor aqueous solubility (0.02 mg/mL),^13^ insufficient chemical stability, short biological half-life (0.7 h^14^ to 2.3 h^8^), and rapid plasma elimination rate. There was only a 0.40 or 10.67% oral bioavailability of SCU in rats and dogs, respectively.^15,16^ Its major metabolites are isoscutellarin (scutellarein-6-*O*-glucuronide) and 6,7-diglucuronide of scutellarein, which are excreted via the bile and urine.^17,18^ To circumvent SCU’s limitations, it has been loaded into nanoformulations of bovine serum albumin,^14^ chitosan,^19−21^ and cyclodextrin,^20−23^ where enhanced solubility, increased stability, and prolonged blood circulation was successfully obtained. However, the efficacy of these SCU-loaded NPs in treating ischemic diseases, such as myocardial or cerebral ischemia, is yet to be evaluated.

PLGA is a copolymer of poly(lactic acid) (PLA) and poly(glycolic acid) (PGA). Many factors have made it a desirable drug carrier for the drug delivery system, including its biocompatibility, biodegradability, established formulation techniques, and ease of processing.^24,25^ Several PLGA microparticle-based products have been approved by the U.S. Food and Drug Administration (FDA),^25,26^ such as Lupron Depot (leuprolide acetate), Trelstar (triptorelin pamoate), and Bydureon Bcise (exenatide). In the present work, SCU-loaded PLGA NPs (SCU-PLGA NPs) were prepared by nanoprecipitation, followed by *in vitro* drug release studies, pharmacokinetics, brain distribution, and therapeutic efficacy investigations in a transient middle cerebral artery occlusion rat model. Promisingly, the superior protective activity of our SCU-PLGA NPs compared to that of free SCU against I/R injury was confirmed via a set of histopathological examinations and neurological function assessments.

## 2 Materials and Methods

### 2.1 Materials

SCU (>98% purity, CAS: 27740-01-8) was supplied by Laizhang Pharmaceutical Technology Co., Ltd. (Kunming, China). A 50:50 dl-lactide/glycolide copolymer (PLGA, 0.2 dL/g, mol. wt. 17,000 g/mol, acid terminated) was kindly gifted by Corbion (Gorinchem, Netherlands). Poly(ethylene glycol) methyl ether-*block*-poly(lactide-*co*-glycolide) (PEG-PLGA, PEG average *M*_n_ 2000, PLGA average *M*_n_ 11,500), poly(vinyl alcohol) (PVA, *M*_w_ 13,000–23,000, 87–89% hydrolyzed), and 2,3,5-triphenyltetrazolium chloride (TTC) were purchased from Sigma-Aldrich (St. Louis, USA). Methanol (analytical grade), acetonitrile (HPLC grade), and acetone (HPLC grade) were purchased from Sinopharm Chemical Reagent Co., Ltd (Shanghai, China). The hematoxylin–eosin staining kit (HE), 4′,6-diamidino-2-phenylindole (DAPI), and dialysis bag (MWCO = 8,000–14,000 Da) were obtained from Solarbio Life Sciences (Beijing, China). The TUNEL apoptosis assay kit was purchased from Roche (Shanghai, China). Ultrapure water was used throughout the experiments.

### 2.2 Preparation of SCU-PLGA NPs

SCU-PLGA NPs were prepared by the nanoprecipitation method.^27^ Briefly, 6 mg of SCU was dissolved in 1.5 mL of methanol by ultrasonication (250 W) at room temperature for 10 min until a yellow saturated solution is formed. PLGA (12 mg) and the PEG-PLGA (8 mg) polymer were dissolved in 3 mL of acetonitrile and mixed with the saturated SCU solution as the organic phase. During continuous stirring, the organic phase was slowly injected into 6 mL of aqueous phase that contained 5% (w/v) PVA. Following evaporation of the organic phase by magnetic stirring for 4 h, the obtained suspension was dialyzed (MWCO = 8,000–14,000 Da, Solarbio Life Sciences, Beijing, China) overnight at room temperature.

### 2.3 NP Characterization

The mean particle size, polydispersity index (PDI), and ζ potential of the NPs were characterized using a Nanobrook 90Plus PALS particle size analyzer (Brookhaven Instruments, New York, USA). For size measurements, each sample was diluted to a suitable concentration (1:40) with ultrapure water and then transferred to a disposal plastic cuvette. Analysis was performed at 25 °C. To measure the surface charge, the samples were diluted with 10 mM sodium chloride solution (1:40). Each sample was measured three times at 25 °C and 12 times per cycle. The particle size and surface charge measurements were determined in triplicate and recorded as their mean values ± standard deviation (SD).

Morphology elucidation was carried out using JEOL JEM2100 transmission electron microscopy (TEM). The NP dispersions were dropped on a carbon-coated 200 mesh copper grid. The TEM images were acquired when the microscopy was operated at 200 kV.

### 2.4 HPLC Analysis of SCU

SCU quantifications were conducted using a Thermo Scientific UltiMate 3000 high-performance liquid chromatography system (Germering, Germany) with an Agilent ZORBAX Eclipse XDB-C18 column (4.6 × 150 mm, 5 μm). The mobile phase consisted of 0.05% phosphoric acid and acetonitrile (15:85) at a flow rate of 1 mL/min. SCU was measured by UV detection at 335 nm, and the column temperature was set at 40 °C. The injection volume was 10 μL by an autosampler. According to the International Council for Harmonisation of Technical Requirements for Pharmaceuticals for Human Use (ICH) guidelines, the method was validated for the SCU assay with respect to specificity, linearity (*R*^2^ > 0.999), accuracy (recoveries between 94.5% and 107.0%), and precision (intraday RSD < 1.28% and interday RSD < 1.91%).

### 2.5 Encapsulation Efficiency and Drug Loading of SCU-PLGA NPs

SCU-PLGA NPs (100 μL) before and after the dialysis were diluted by 900 μL of methanol and sonicated for 30 min to solubilize the NPs and release SCU. After centrifugation at 10,000 rpm (7583×*g*), the SCU concentration was determined by the HPLC method. The encapsulation efficiency (EE) and drug loading (DL) were calculated according to the following equations, respectively
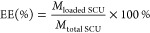
1
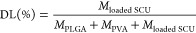
2where *M*_total__SCU_ is the mass of the total SCU content before dialysis, *M*_loaded SCU_ is the mass of the SCU content loaded in the NPs after dialysis, *M*_PLGA_ is the mass of the total PLGA and PEG-PLGA polymer content added in the system, and *M*_PVA_ is the mass of the total PVA content added in the system.

### 2.6 *In Vitro* Drug Release Study

The method was similar to that of Mehrotra and Pandit.^28^ Free SCU or SCU-PLGA NPs (1 mL) dispersed in PBS (pH 7.4) were placed in a dialysis bag (Solarbio Life Sciences, MWCO = 8000–14,000 Da, 25 mm). Then, the dialysis bag was placed in a 50 mL centrifuge tube containing 20 mL of PBS solution (pH 7.4) with 2 mg/mL EDTA-2Na (disodium ethylenediaminetetraacetate, a metal ion chelating agent) and incubated in a water bath shaker at 37 °C. The sample (1 mL) was drawn out from the centrifuge tube at 0.5, 2, 4, 6, 8, 12, 24, and 48 h. Fresh PBS solution (1 mL) at 37 °C was added to the centrifuge tube after each sampling.

### 2.7 Animals

SPF-grade healthy male Sprague-Dawley (SD) rats (260 ± 20 g, 9–11 weeks) were obtained from Changsha Tianqin Biotechnology Co., Ltd. with the certificate number SCXK (Xiang) 2014–0011 or SCXK (Xiang) 2019–0014. Rats were housed at a constant room temperature of 22 °C under a 12 h light–dark cycle with free access to food and water. All experiments were carried out following the criteria outlines in the Guide for the Care and Use of the Animal Management Rules of the Health Ministry of the People’s Republic of China (documentation number 55, 2001, China) and the protocol (no. 1801215) approved by the Experimental Animal Ethics Committee of Guizhou Medical University.

### 2.8 Transient Rat Middle Cerebral Artery Occlusion Model

An intraluminal suture technique was used to induce transient rat middle cerebral artery occlusion (MCAO).^29,30^ Briefly, an intraperitoneal injection of 10% chloral hydrate (400 mg/kg) was used to anesthetize 12 h fasted rats. The anesthetized rats were placed on a sterile surgery table and immobilized in the supine position on the rat fixture. The rat neck was disinfected with 75% alcohol. In a midline incision of the neck, the left common carotid artery (CCA), external carotid artery (ECA), and internal carotid artery (ICA) were isolated. In order to block the origin of the middle cerebral artery (MCA), a 45 mm long monofilament nylon suture (*d* = 0.38 ± 0.02 mm, 2838-A4, Beijing Sinotech Co. Ltd) with its tip rounded was introduced into the ECA lumen and advanced into the ICA for 18 mm. During the surgical procedure, the body temperatures of the rats were maintained at 36.5–37.5 °C by an infrared heat lamp. Sham-operated animals did not receive I/R. The nylon suture was removed after 60 min of ischemia to establish reperfusion.

### 2.9 Pharmacokinetic Study

After MCAO reperfusion, 12 SD rats (weighing 250 ± 20 g) were immediately divided randomly into two groups: SCU (3.5 mg/kg) and SCU-PLGA NPs (SCU: 3.5 mg/kg and PLGA: 17.74 mg/kg). The samples were injected intravenously (via tail vein) once a day for 3 days. The rat blood samples (0.25 mL) were collected at 0.083, 0.167, 0.333, 0.5, 1, 4, 8, and 12 h after the last administration via caudal vein into centrifuge tubes coated with 1% heparin. According to the Technical Guidelines for Non-Clinical Pharmacokinetic Studies of Drugs in China, the amount of blood samples from each rat within 24 h should not exceed 2 mL. Therefore, each rat group (*n* = 6) was divided into two groups (*n* = 3), enabling collecting alternating blood sampling, so the total blood samples collected from a single animal within 24 h did not exceed 10% of its body weight. The collected blood was centrifuged at 3500 rpm (928×*g*) for 10 min at 4 °C. The supernatant (100 μL) was transferred to the centrifuge tube. Puerarin (50 μL) in methanol (12.04 ng/mL) as the internal standard, 50 μL of 1% formic acid as the water solution, and 400 μL of methanol were added in order, mixed for 2 min, sonicated for 10 min, and centrifuged at 12,000 rpm (10,920×*g*) for 10 min at 4 °C. The supernatant (500 μL) was collected and evaporated under a nitrogen gas flow at 37 °C. The residue was reconstituted in 200 μL of 50% methanol and centrifuged at 14,000 rpm (21,475×*g*) for 10 min at 4 °C. The supernatant was transferred into the sampler vial, and 1 μL was subsequently injected for UPLC–MS analysis.

### 2.10 Brain Distribution

The MCAO rats treated with SCU or SCU PLGA NPs were sacrificed by cervical dislocation at 4 h after the last tail vein injection of free SCU and SCU PLGA NPs. The brain was quickly removed and homogenized with saline. Each tissue homogenate (200 μL) was placed in a 2 mL centrifuge tube. Then, 50 μL of 30.1 ng/mL puerarin, 50 μL of 1% formic acid water, and 800 μL of methanol were added in the tube. The homogenate mixture was vortexed, mixed for 30 s, sonicated for 15 min, and centrifuged at 12,000 rpm (10,920×*g*) at 4 °C for 10 min. The supernatant (950 μL) was dried under nitrogen at 37 °C. The residues were resuspended by 400 μL of 50% methanol, and 1 μL of supernatant was injected for UPLC-MS analysis.

### 2.11 UPLC–MS Analysis

The UPLC–MS measurement for SCU was reported by Li et al.^31^ An Acuity UPLC system equipped with a binary pump, degasser, autosampler, and temperature-controlled column compartment and a TQD quantum triple-quadrupole mass spectrometer equipped with an electrospray ionization (ESI) source (Waters Corp., Manchester, UK) were used for SCU analysis in the pharmacokinetics and tissue distribution studies. Liquid chromatography (LC) was performed by a Waters Van Guard BEH C18 column (2.1 mm × 50 mm, 1.7 μm) at 45 °C with the mobile phase consisting of 0.1% formic acid in acetonitrile (A) and 0.1% formic acid water (B). The gradient program is as follows: 0–0.5 min, 5% A and 95% B; 0.5–3 min, 5–95% A and 95–5% B; and 3–3.5 min, 95–5% A and 5–95% B. The peaks were obtained at a flow rate of 0.3 mL/min with a sample injection volume of 1 μL. The electrospray positive ionization (ESI^+^) was used for detection and analysis. In the positive ion mode, the SCU parameters are as follows: capillary voltage at 3 kV, cone voltage at 30 V, and collision energy at 20 eV; the puerarin parameters are as follows: capillary voltage at 3 kV, cone voltage at 40 V, and collision energy at 30 eV. SCU and puerarin (internal standard) were quantified using the selected ion recording mode (SIR) of their parent ions, 463 and 417, respectively.

### 2.12 Pharmacodynamic Treatment

Immediately after MCAO reperfusion, the rats were randomly divided into four groups: MCAO + saline, MCAO + blank PLGA NP group (PLGA: 17.74 mg/kg/day), MCAO + free SCU group (SCU: 3.5 mg/kg/day), and MCAO + SCU-PLGA NP group (SCU: 3.5 mg/kg/day; PLGA: 17.74 mg/kg/day). Free SCU was only dissolved in PBS solution before the injection. The nanosuspensions containing SCU-PLGA NPs or blank PLGA NPs were obtained by ultrafiltration centrifugation using freshly prepared NPs. The rats were treated intravenously (via tail vein) with 2 mL/kg PBS solution or nanosuspension once a day for 3 days. Untreated rats and sham-operated rats were injected with saline and used as controls. The sham-operated rats received the same procedure as those in the MCAO group, but the middle cerebral artery was not sutured.

### 2.13 Neurological Evaluations

After 24 h of reperfusion, the rat neurological function was evaluated blindly according to the Longa score method.^32^ The neurological findings were scored on a five-point scale: 0—no neurological deficit (normal); 1—failure to extend contralateral forepaw (mild); 2—spin longitudinally (moderate); 3—falling to the contralateral side (severe); and 4—no spontaneous walking with a depressed level of consciousness (very severe). The rats with the neurological deficit score 1–3, which indicated the successful establishment of the rat MCAO model, can be included in the subsequent experiments.

### 2.14 Change of Bodyweight

All rats were weighed before surgery and on day 3 after treatment. The weight variation after ischemia of each animal was evaluated with respect to its own pre-ischemia weight. Bodyweight changes were expressed as the percentage of the baseline value obtained prior to surgery.

### 2.15 TTC Assessment of Infarct Area

The infarct area after MCAO and following the SCU, PLGA NP, or SCU-PLGA NP treatment was evaluated by the 2,3,5-triphenyltetrazolium chloride (TTC) staining method. The rats were sacrificed at 24 h after the third dose. The brain was isolated and placed on ice. The cerebellum and olfactory bulb part of the brain was removed, and the whole brain was rinsed quickly with 0.9% saline. After absorbing the excess water on the surface of the brain tissue by a filter paper, the brain was frozen at −20 °C for 45 min. The brain was then divided into five sections of 2 mm thickness. TTC (Sigma Aldrich, USA) staining was used to quantify the cerebral infarction. All slices were incubated for 15 min in a 5 mL 1% solution of TTC at 37 °C and were turned around every 5 min to make the brain tissue evenly stained. The cerebral normal area appeared red, while the cerebral infarcted area appeared whitish. The stained brain slices were fixed by immersion in 5 mL 4% formaldehyde solution. Infarct areas in each section were measured using Image J software, and the cerebral infarct area was calculated with the following formula
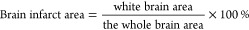
3

### 2.16 Hematoxylin and Eosin Staining

Brain tissues were obtained in each group according to the procedure described in the TTC assessment of infarct area. All these fresh brain tissues were embedded in a wax block after being placed and fixed in 50 mL of 10% formaldehyde solution for at least 24 h. Afterward, all brain tissues were cut into thin slices of 3 μm thickness, fixed on glass slides, and dried for staining. As instructed, they were immersed in xylene and ethanol at gradient concentration, stained with hematoxylin and eosin, and then sealed with resin. The slices were left to air dry and then imaged using an optical microscope. The pathological morphology changes of cerebral ischemia in the hippocampus and cortex of rats in each group were observed.

### 2.17 TUNEL Assay

The cut brain tissues fixed with paraffin were sliced; dewaxed with xylene (15 min × 2 times); dehydrated with 100% (5 min × 2 times), 85% (5 min × 1 times), and 75% ethanol (5 min × 1 times), respectively; and washed with the ultrapure water. Next, the slices were incubated with protein kinase K for 25 min at 37 °C. After washing with phosphate-buffered saline (PBS, pH 7.4) three times, each time 5 min, the terminal deoxynucleotidyltransferase (TdT) and fluorescein isothiocyanate (FITC)-labeled dUTP (1:9) solution was added and stained with these slices for 2 h at 37 °C. Following three rounds of washing with PBS, the 4′,6-diamidino-2-phenylindole (DAPI) solution was added and incubated for 10 min at room temperature in the dark. Subsequently, the slices were washed with PBS again (5 min × 3 times). After the nucleus was stained with DAPI (UV excitation wavelength 330–380 nm and emission wavelength 420 nm) and FITC (excitation wavelength 465–495 nm and emission wavelength 515–555 nm), the nucleus appeared with blue and the apoptotic cells were stained with green. Three rats in each group were chosen. Three fields of view were imaged from each rat and counted under a Nikon Eclipse C1 fluorescence microscope. Apoptotic cells (green fluorescence) and other normal cells (blue fluorescence) in each section were measured using Image-Pro Plus 6.0 software (Media Cybernetics, Inc., Rockville, MD, USA), and the apoptotic cell was calculated using the following formula
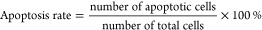
4

### 2.18 Statistical Analysis

The pharmacokinetic results were analyzed by a WinNonLin 8.2 software. SPSS 17.0 was used to analyze all the experimental data. For the pharmacokinetic results, independent samples *t*-test was used. Kruskal–Wallis non-parametric test (K–W test) was used in the statistical analysis of neurological deficit score. Cerebral infarction area, the change in bodyweight, and neuronal apoptosis were analyzed using one-way analysis of variance (ANOVA), followed by Dunnett T3 test. Data were expressed as mean ± SD of at least three independent experiments. *P* values < 0.05 were considered as statistically significant.

## 3 Results

### 3.1 Preparation and Characterization of SCU-PLGA NPs

**Figure 1:**
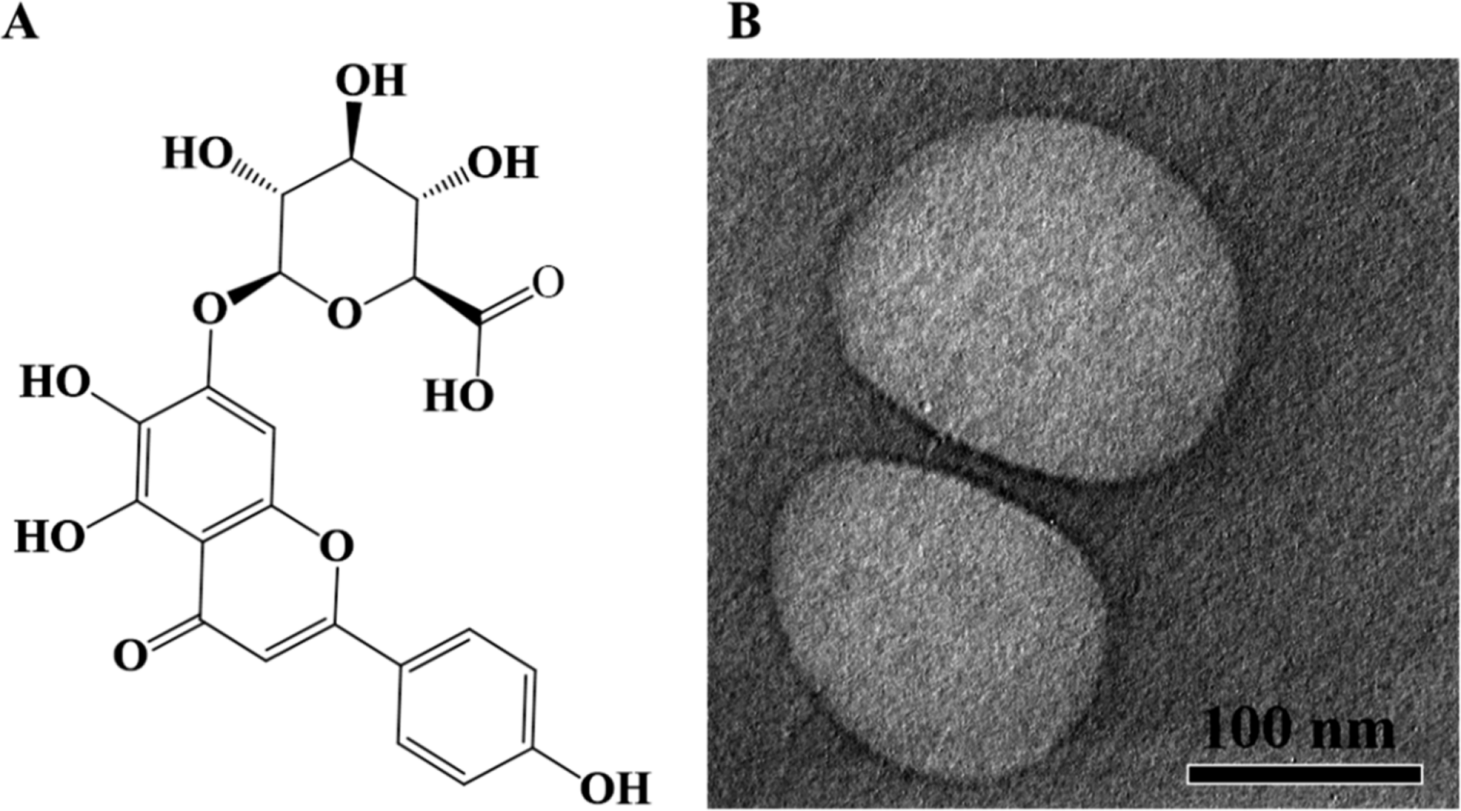
Chemical structure of SCU (A) and (B) TEM structural elucidation of SCU-PLGA NPs.

SCU (Figure 1A) contains three phenolic hydroxyl groups, two of which are in the *ortho*-phenolic hydroxyl group and are easily oxidized, thus resulting in poor stability.^33^ To overcome such an issue, SCU-PLGA NPs were prepared using the nanoprecipitation method. Their hydrodynamic size was 187.89 ± 3.42 nm, with a low PDI of 0.077 ± 0.031 and a slightly negative surface charge (the ζ potential was −6.99 ± 1.75 mV). SCU-PLGA NPs exhibited an oval morphology following TEM examination (Figure 1B).

The stability of SCU-PLGA NPs was assessed. SCU-PLGA NP size, EE, and DL were determined. As indicated in Table 1, the size and PDI of SCU-PLGA NPs at 4 °C did not change over 1 week. More importantly, the SCU EE and DL were stable at 63.63 ± 4.41 and 1.19 ± 0.09%, respectively.

**Table 1:** Stability of SCU-PLGA NPs Stored at 4 °C for 1 Week^a^

days	0	1	2	7
*Z*-ave ± SD (nm)	187.89 ± 3.42	188.45 ± 2.87	186.72 ± 5.87	209.36 ± 10.55
PDI ± SD	0.077 ± 0.031	0.100 ± 0.018	0.102 ± 0.015	0.109 ± 0.016
ζ potential ± SD (mV)	–6.99 ± 1.75	–7.49 ± 0.94	–15.11 ± 2.16	–1.95 ± 1.05
EE (%)	63.63 ± 4.41	62.45 ± 3.67	63.67 ± 5.71	66.04 ± 6.75
DL (%)	1.19 ± 0.09	1.17 ± 0.07	1.19 ± 0.13	1.24 ± 0.10

a The hydrodynamic size, PDI, ζ potential, EE, and DL were measured using dynamic light scattering (DLS) or HPLC analysis. Data shown as mean ± SD (*n* = 3).

### 3.2 SCU-PLGA NPs Increased the SCU Stability and Sustained Its Release

**Figure 2:**
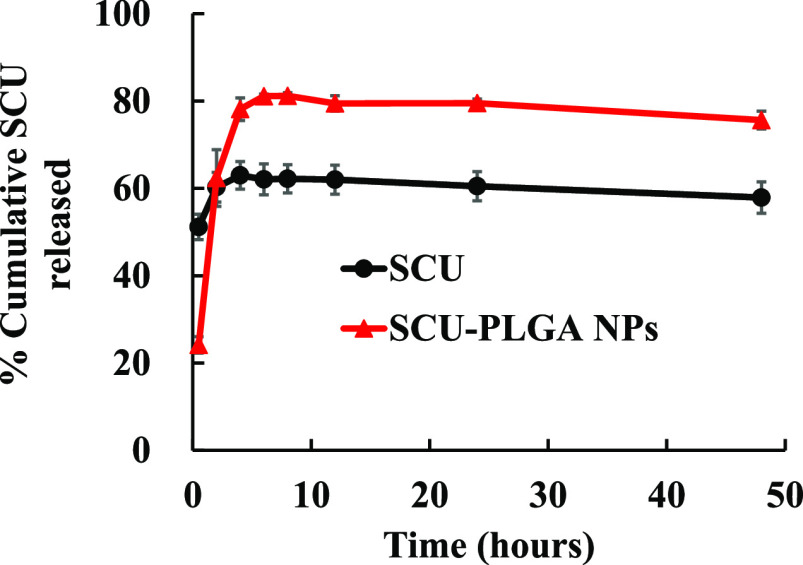
Drug release profile of SCU-PLGA NPs at 37 °C in PBS (pH 7.4) with 2 mg/mL EDTA-Na2. SCU or SCU-PLGA NPs (1 mL) were placed in a dialysis membrane and placed in 20 mL of PBS media with 2 mg/mL EDTA-2Na at 37 °C. The sample was collected from the external medium at 0.5, 2, 4, 6, 8, 12, 24, and 48 h, and the cumulative SCU released was quantified by the HPLC method at 335 nm. Data was shown as mean ± SD (*n* = 3).

Figure 2 depicts the release profile of SCU-PLGA NPs in PBS with 2 mg/mL EDTA-2Na as a stabilizer at 37 °C. During the first 4 h of dialysis, SCU-PLGA NPs showed a fast initial release, followed by a prolonged release over 44 h. On the contrary, nearly 50% of free SCU was released in the first 0.5 h. The lag phase of SCU-PLGA NPs lasted longer than that of free SCU, indicating improved SCU stability following PLGA encapsulation. The biphasic SCU-PLGA NP release profile with an initial rapid burst of SCU release attributed to the surface-adsorbed drug, followed by a slower release as the entrapped SCU diffuses from the PLGA NPs to the release medium.^24,34^ However, 100% SCU from the PLGA NPs release could not be achieved, properly due to the lower stability of the released SCU in solution, where the anticipated continuous increase in the cumulative SCU released was masked by SCU degradation/oxidation in the external medium.

### 3.3 SCU-PLGA NPs Increase the SCU Level in the Blood and Brain of Rats with Cerebral Ischemia

**Figure 3:**
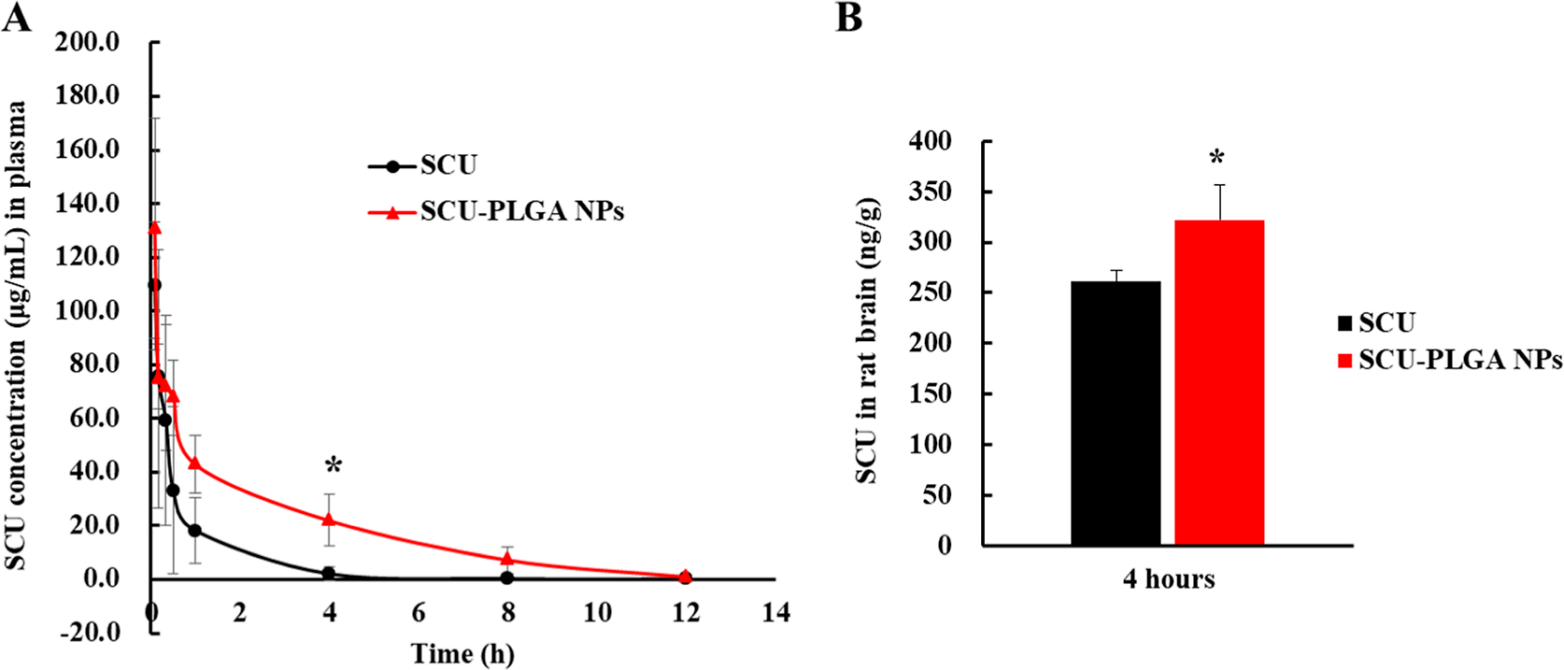
Plasma profile and brain level of intravenously administered SCU-PLGA NPs and free SCU in MCAO rats. (A) After MCAO operation, SCU (3.5 mg/kg) and SCU-PLGA NPs (SCU: 3.5 mg/kg; PLGA: 17.74 mg/kg) were injected intravenously (via tail vein) once a day for 3 days. The rat blood samples were collected at 0.083, 0.167, 0.333, 0.5, 1, 4, 8, and 12 h after the last administration via caudal vein, and the amount of SCU in the rat plasma was quantified by the HPLC-MS method. (B) SCU brain level in rats treated as described in (A) and collected 4 h after the last dosing. Data shown as mean ± SD (*n* = 3). Statistical analysis of free SCU vs SCU-PLGA NPs was determined using the independent samples *t*-test by SPSS software 17.0, *P < 0.05.

The *in vivo* performance of the developed SCU-PLGA NPs in a cerebral ischemic model was studied using the MCAO rat model. Immediately following MCAO reperfusion, the rats were injected intravenously with SCU or SCU-PLGA NPs at a SCU dose of 3.5 mg/kg for three consecutive days. Blood was collected at different time points from the last dose, and SCU was extracted from the blood and quantified using UPLC–MS. Figure 3A and Table 2 depict the plasma profile and pharmacokinetic parameters of SCU-PLGA NPs and free SCU in the MCAO rat model, respectively. SCU-PLGA NPs exhibited a rapid blood clearance in the first 0.083 h (5 min) post-injection, which was comparable to free SCU. It is probably due to the free drug adsorbed to the surface of SCU-PLGA NPs, which was consistent with the observation of *in vitro* drug release. However, from 0.333 h (20 min) to 12 h, the plasma concentration of SCU in the SCU-PLGA NP group was higher than that of free SCU, which implied that the administration of SCU-PLGA NPs provided a higher SCU concentration in the systemic circulation (mean plasma concentration: 20.83 ± 1.46 vs 7.27 ± 1.46 μg/mL) and for an extended period of time (mean retention time MRT_0–∞_: 2.75 ± 0.31 vs 1.03 ± 0.15 h). This was further supported with a 2.9-fold increase (*P* < 0.01) in the AUC of SCU-PLGA NPs compared to the free SCU (249.95 ± 17.54 vs 87.23 ± 52.28 h × μg/mL) and a 1.5-fold extension (*P* < 0.05) in the half-life time of SCU (1.91 ± 0.34 vs 1.31 ± 0.12 h). These are in agreement with other reports that revealed extended drug blood circulation following NP encapsulation.^35^ In addition, the extended blood circulation of our SCU-PLGA NPs resulted in a significantly higher SCU brain level (321.87 ± 35.09 ng/g) than free SCU (260.97 ± 11.59 ng/g) in rats with cerebral ischemia at 4 h after last injection (Figure 3B, P < 0.05).

**Table 2:** Pharmacokinetic Parameters of Free SCU and SCU-PLGA NPs in MCAO Model Rats (*n* = 3)^a^^,^^b^

parameters	unit	free SCU	SCU-PLGA NPs
AUC_0–*t*_	h*μg/mL	87.23 ± 52.28	249.95 ± 17.54**
AUC_0–∞_	h*μg/mL	87.65 ± 52.26	252.70 ± 17.71**
*C*_max_	μg/mL	109.4 ± 23.9	130.8 ± 40.9
CL	L/h/kg	48.74 ± 22.25	13.89 ± 0.95
Vz	L/kg	93.21 ± 47.01	38.3 ± 7.81
*t*_1/2_	h	1.31 ± 0.12	1.91 ± 0.34*
MRT_0–∞_	h	1.03 ± 0.15	2.75 ± 0.31**

a Statistical significance was assessed using the independent samples *t*-test for comparisons between groups by SPSS 17.0. *P* values < 0.05 were considered as statistically significant. * denotes the comparison between control and the treatments. (*P < 0.05 and **P < 0.01 compared with free SCU).

b AUC_0–*t*_, area under plasma concentration versus time curve from zero to last sampling time; AUC_0–∞_, area under plasma concentration versus time curve from zero to infinity; *C*_max_, maximum plasma concentration; CL, total body clearance; Vz, apparent volume of distribution; *t*_1/2_, terminal elimination half-life; and MRT_0–∞_, mean retention time from zero to infinity. The pharmacokinetic results were analyzed by WinNonLin 8.2 software. Data were expressed as mean ± SD of three independent experiments.

### 3.4 Intravenously Administered SCU-PLGA NPs Rescue Cerebral Ischemia in Rats and Improved Rat Behavior

**Figure 4:**
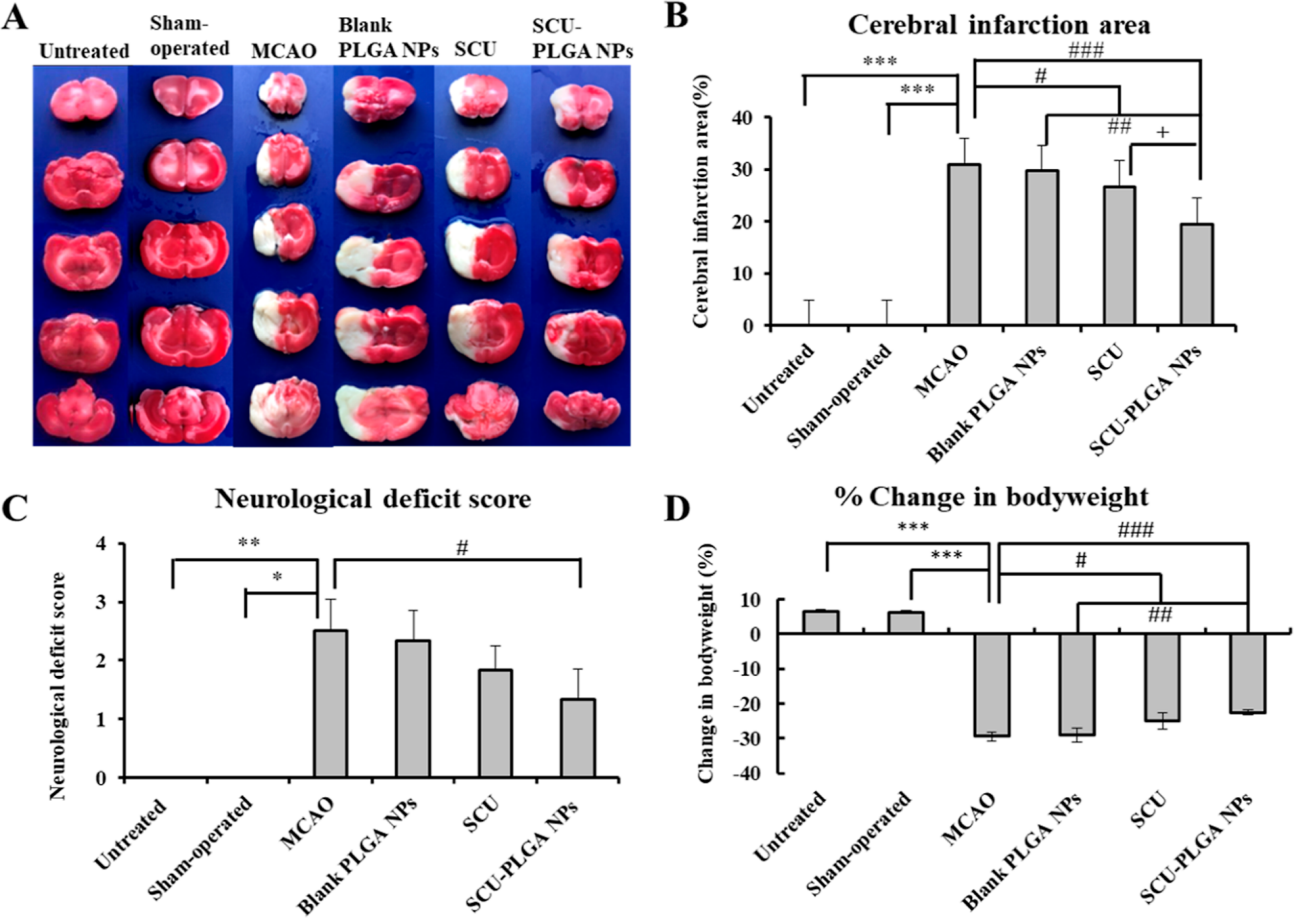
Intravenously administered SCU-PLGA NPs rescued cerebral ischemia in rats and improved the rat behavior. Brain infarct, neurological deficits and bodyweight change of rats at 24 h after administering the different treatments. (A) Representative images of TTC-stained brain slices (all five slices in one rat brain, *n* = 1); (B) cerebral infarction area analysis; (C) neurological deficit scores in rats; and (D) post-operative bodyweight change in rats. Statistical analysis was performed using K–W test for neurological deficit score and one-way analysis of variance (ANOVA), followed by Dunnett’s T3 test for cerebral infarction area and the change in body weight. Compared with the untreated or sham-operated groups, *P < 0.05, **P < 0.01, and ***P < 0.001; compared with the MCAO group or blank PLGA NPs, ^#^*P* < 0.05, ^##^*P* < 0.01, and ^###^*P* < 0.001; and compared with SCU, ^+^*P* < 0.05. Data shown as mean ± SD (*n* = 6).

To assess the capability of SCU-PLGA NPs in rescuing cerebral ischemia, the MCAO model was first established in rats. Immediately following MCAO reperfusion, the rats were injected intravenously with ∼520 μl (2 mL/kg) of PBS solution containing SCU (3.5 mg/kg), SCU-PLGA NPs (SCU: 3.5 mg/kg; PLGA: 17.74 mg/kg), or blank PLGA NPs (PLGA: 17.74 mg/kg). Untreated and sham-operated rats were used as controls. After 24 h of the last dosing, the brain was isolated, sliced, and stained with 2,3,5-triphenyltetrazolium chloride (TTC) (Figure 4A). In contrast to untreated and sham-operated rats, the MCAO rats had white infarctions in the ipsilateral hemispheric brain slices (Figure 4A), indicating the successful establishment of the MCAO model in the brain. Diseased rats injected with free SCU or SCU-PLGA NPs showed reduced infarct areas, which were the least pronounced in the SCU-PLGA NP group. On the other hand, the area of cerebral infarction in rats injected with blank PLGA NPs was not significantly reduced, confirming the SCU activity against cerebral infarction.

To provide some quantitative data, the infarcted areas in each section of the whole brain were measured using Image J software shown in Figure 4B. The cerebral infarct area volume in the MCAO model group was about 31%, which is in agreement with other studies.^36−39^ As expected, rats injected with the blank PLGA NPs showed cerebral infarct volume comparable to the saline-treated group, confirming that PLGA NPs alone did not have any therapeutic effect on cerebral infarction. On the contrary, a significant reduction was noticeable in the infarcted areas of diseased rats treated with SCU-PLGA NPs (19%) and free SCU (27%), confirming the superiority of SCU-PLGA NPs in rescuing cerebral ischemia in rats. The higher efficacy of our SCU-PLGA NPs could be attributed to the prolonged blood circulation of our SCU-PLGA NPs, resulting in higher SCU levels in the brain (Figure 3B) with a sustained release profile (Figure 2 and Table 2). Next, a behavioral study was conducted using a Longa scoring method to assess if the reduction in cerebral ischemia affects the rats’ behavior. Interestingly, diseased rats treated with saline or blank PLGA NPs exhibited comparable neurological scores of 2.50 ± 0.55 and 2.33 ± 0.52, respectively. On the contrary, both SCU groups showed better performance since lower neurological scores were recorded in the diseased rats treated with SCU-PLGA NPs (1.33 ± 0.52) (*P* < 0.05) and free SCU (1.83 ± 0.41) (Figure 4C).

More importantly, a significant drop (∼29%) was detected in the bodyweight of the diseased rats treated with saline or blank PLGA NPs up to 3 days of post-operation. A lower reduction in the bodyweight of the diseased rats treated with SCU-PLGA NPs and free SCU (23 vs 25%, respectively) (*P* < 0.05 compared to the MCAO group) (Figure 4D) consistently confirms the preserved efficacy of SCU-PLGA NPs in rescuing cerebral ischemia in rats.

### 3.5 SCU-PLGA NPs Reverse Histopathological Changes in Cerebral Ischemic Brain

**Figure 5:**
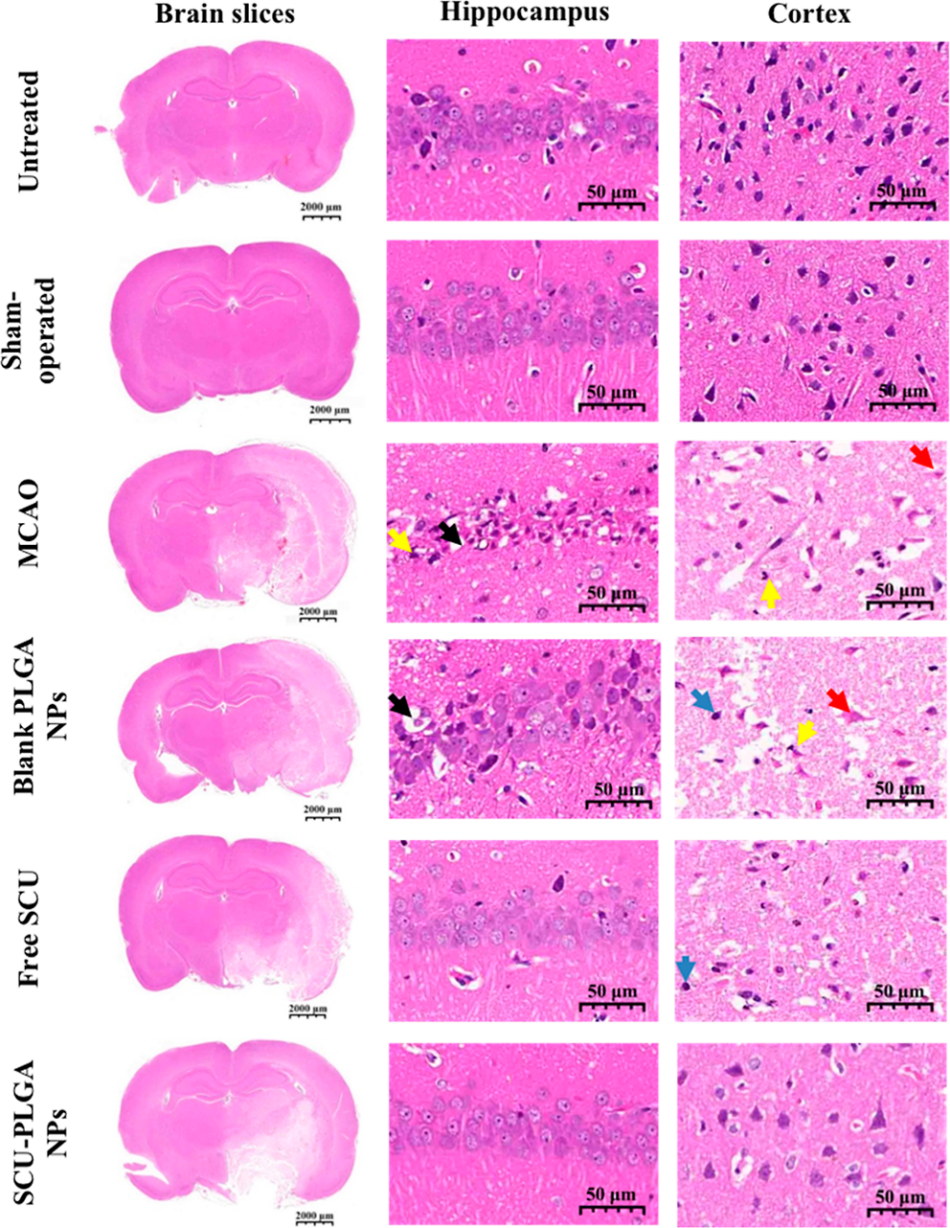
SCU-PLGA NPs reversed histopathological changes in brain tissues following cerebral ischemia. Representative micrographs of H&E-stained brain slices (scale bar: 2000 μm), hippocampus and cortex (scale bar: 50 μm) of untreated or sham-operated rats, and MCAO rat models treated with saline (control), blank PLGA NPs, free SCU, and SCU-PLGA NPs. Black arrows represented the vacuoles; yellow arrows represented the nuclear pyknosis or fragmentation; blue arrows represented a little lymphocyte infiltration; and red arrows represented the enhancement of eosinophilic of neurons.

Figure 5 shows the histology of cerebral ischemia/reperfusion injury regions located in the hippocampus and cortex areas of the brain. The lighter hematoxylin and eosin (H&E) brain tissue staining identified in the infarcted cerebral areas of the MCAO rat model was indicative of liquefactive necrosis.^40^ In the hippocampus region, no histological abnormalities were observed in the untreated and sham-operated rats, while the saline-treated MCAO model group showed that the majority of neurons in the infarct core appeared shrunken and contained triangulated pyknotic nuclei (yellow arrows) with many vacuoles (black arrows) in the neuron space. The shrunken neurons and some vacuoles were also observed in the hippocampus of the blank PLGA NP group. However, unlike the cell apoptosis observed in the MCAO model group, a large number of swollen cells were seen in the blank PLGA NP group, which requires further investigation. As ischemia deprives the cells of energy substrates, the sodium pump fails, leading to ATP depletion, which causes swelling of the cell, which may eventually rupture the plasma membrane.^41−43^ On the other hand, a tight arrangement with single neurons shrinking similar to the sham-operated group appeared in the SCU and SCU-PLGA NP groups, confirming that SCU indeed reversed the brain damage induced by the MCAO model. In the cortex area, the tissue structure in the untreated and sham-operated groups was normal and uniform. No obvious degeneration, inflammation, and necrosis of neurons were observed. Necrosis and nuclear fragmentation (yellow arrows) and eosinophilic cytoplasm (red arrows) were seen in the cortex, accompanied by a small amount of lymphocyte infiltration (blue arrows) in the MCAO, blank PLGA NP, and SCU groups. Neurons with rare neuronal necrosis and a relatively regular morphology in the SCU-PLGA NP group were observed, confirming that SCU-PLGA NPs are more effective than free SCU in treating cerebral rat ischemia.

### 3.6 SCU-PLGA NPs Reduce Neuronal Apoptosis following Cerebral Ischemia

**Figure 6:**
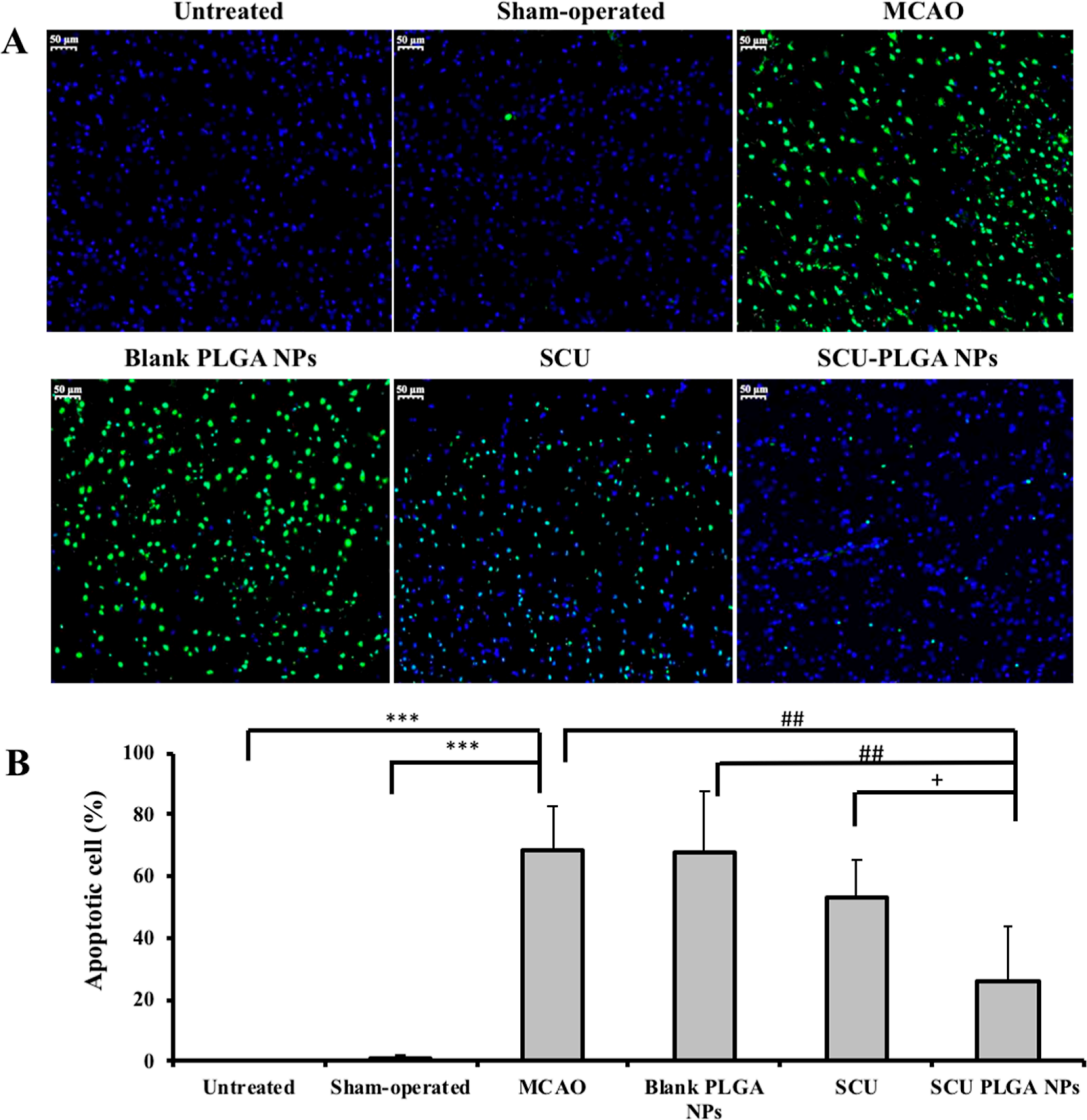
SCU-PLGA NPs reduced neuronal apoptosis following cerebral ischemia. (A) Representative images of cells stained with TUNEL. The nucleus was stained with DAPI (blue), and the apoptotic cells were stained with TUNEL (green). Scale bar: 50 μm. (B) Percentage of apoptotic cells in the brain of MCAO rats. SD rats were divided into untreated, sham-operated, MCAO (saline), blank PLGA NP, SCU, and SCU-PLGA NP groups (*n* = 3). Three sections from one rat were used for the TUNEL assay. The ischemic brain analysis from three sections of three rats in one group was carried out using Image-pro plus 6.0 (Media Cybernetics, Inc., Rockville, MD, USA). All the results are expressed as average ± SD (*n* = 3). Statistical analysis was performed using ANOVA, followed by Dunnett’s T3 test. Compared with the untreated group or the sham-operated group, ***P < 0.001; compared with the MCAO model or blank PLGA NPs, ^##^*P* < 0.01; compared with SCU, ^+^*P* < 0.05.

Following H&E histological examination, cell apoptosis in brain slices was confirmed using TUNEL staining, as shown in Figure 6A. TUNEL-stained brain sections obtained from the sham-operated group showed a few TUNEL-positive cells, whereas a significantly higher number of TUNEL-positive cells were observed in the ischemic hemisphere of saline- or blank PLGA NP-treated diseased rats. Promisingly, the number of TUNEL-positive cells was markedly decreased in the diseased rats treated with SCU or SCU-PLGA NPs. Figure 6B depicts the quantitative results of apoptosis in the ischemic cortex from three different sections of three rats per group. As expected, no apoptosis was detected in the normal cerebral cortex area, and single TUNEL-positive cells were occasionally seen in the untreated and sham-operated rats. The percentage of apoptotic cells was only 1.12 ± 1.09%, which indicated minimal injury to the brain tissue during the sham operation. Compared with the untreated and the sham-operated groups, a higher percentage of apoptotic cells (68.44 ± 14.38 and 67.47 ± 19.92%) (*P* < 0.001) in the cortex of the saline- and blank PLGA NP-treated MCAO model group was detected, indicating the widespread of apoptotic cells in the rat brain following the I/R injury.

In agreement with the interesting findings obtained earlier (Figures 4 and 5), the percentage of apoptotic cells in the ischemic brain tissue significantly decreased in SCU- and SCU-PLGA NP-treated groups (53.37 ± 12.09 and 26.28 ± 17.56%, respectively). More importantly, cell apoptosis in diseased rats treated with SCU-PLGA NPs was significantly lower than the saline-treated (*P* < 0.01) and SCU-treated (*P* < 0.05) groups, verifying that SCU-PLGA NPs had a better protective effect against cerebral I/R injury than free SCU at an equivalent dose.

## 4 Discussion

Ischemic brain injury accounts for 88% of stroke incidence.^44^ Thrombolytic therapy is the conventional approach to restoring cerebral blood perfusion.^45^ However, this ischemia/reperfusion (I/R) strategy may further exacerbate tissue damage by causing increased inflammation, oxidative stress, glial activation, as well as excitotoxicity.^4,45^ Therefore, neuroprotective agents are necessary to treat ischemic stroke. In this regard, several TCM-derived drugs have been exploited to treat cerebrovascular diseases and disorders, including breviscapine and SCU, which have anti-inflammatory, antioxidative, and beneficial vascular, and hemodynamic functions, in addition to their low toxicity, low cost, and accessibility.^2−12^ Recently, SCU’s cardioprotective and neuroprotective properties have been explained by three main mechanisms.^2−4^ First, the cardio- and neuroprotective effects of SCU are dependent on its effects on nitric oxide synthases (NOS). Three distinct NOS isoforms have been identified, namely, neuronal nNOS, endothelial eNOS, and inducible iNOS. As a signaling molecule, nitric oxide (NO) regulates numerous biological processes in the brain. Depending on the NOS isoform, it can contribute to either neuroprotection or neurotoxicity during cerebral ischemia. Ischemia causes excessive production of NO by iNOS, which is cytotoxic, while NO produced by eNOS is beneficial (vasodilation, platelet aggregation inhibition, and endothelial adhesion of monocytes). Promisingly, studies have shown that SCU can promote the expression of eNOS and suppress the expression of iNOS.^46^ Thus, SCU can protect the brain from the ischemic injury. Second, SCU is believed to have a protective effect during ischemia reperfusion by inhibiting pro-inflammatory cytokines (TNF-α, IL-1β, IL-6, and IL-8), releasing creatinine kinase, and promoting angiogenesis in the treatment of ischemia-associated tissue damage.^47^ Third, SCU may help to decrease the Ca^2+^ overload, improve the Ca^2+^-ATPase activity, and reduce the excitotoxicity by balancing the imbalance between excitatory and inhibitory amino acids.^48^ In the present work, we have not been able to distinguish the neuroprotection from the vascular protection of SCU; however, this could be elucidated in the future using a series of experiments, including anti-oxidative (SOD, MDA, and ROS), anti-inflammation (TNF-α, IL-1β, and IL-6), and vascular protection (VEGF and NO) markers.

In addition to its low solubility in both water (0.02 mg/mL) and lipid (log *P* = −2.37 in *n*-octanol/phosphate buffer (pH 7.4) at 37 °C),^13^ SCU is subjected to rapid and extensive biotransformation in the liver to hydrophilic aglycone-conjugated metabolites that are excreted by the bile and renal systems, thus lowering the SCU concentration in the systemic circulation.^4^ Therefore, high doses are always required to demonstrate the therapeutic efficacy in vivo.^4^ To date, a range of dosage forms containing breviscapine such as injections, granules, ordinary tablets, dispersible tablets, capsules, mixture, and drop pills are in the market.^2^ Therapeutic breviscapine oral (tablets) and injectable formulations contain relatively high concentrations of SCU (at least 83.5 and 91.0%, respectively) (Chinese Pharmacopoeia Commission, 2020). Breviscapine injections are generally administered at doses of 5 to 20 mg per day at one time for adults (0.1–0.4 mg/kg/day), whereas oral administration is done at dosages of 120 to 240 mg divided into three doses per day.^2^ Studies have shown that encapsulating drugs into NPs protect them from being metabolized by liver enzymes and reduce their renal clearance.^49^ Several attempts have been reported to load SCU into a range of delivery systems, such as hydrophilic cyclodextrin derivatives,^20−23^ amphiphilic chitosan,^19−21^ and positive lipid carriers^7^ to enhance SCU stability and alter its pharmacokinetics *in vivo*. Consistent with others, our novel SCU-PLGA NPs increased SCU stability and extended its blood circulation and brain accumulation (Figure 3).

Numerous reports have been published on the potentials of SCU in treating cardiovascular and cerebrovascular ischemic diseases,^2−4^ but no report yet on SCU-loaded NPs. Interestingly, our study has revealed, and for the first time, the superior protective effect of SCU-PLGA NPs in a transient MCAO rat model against cerebral ischemia/reperfusion injury, as supported by histological examination (Figure 5), TUNEL staining (Figure 6), and neurological deficit scoring (Figure 4A). This could be attributed to the enhanced stability of SCU nanoformulation (Figure 2) combined with higher SCU levels in the plasma and brain (Figure 3), as previously reported with other SCU-loaded NPs.^6−9^

Recently, there are three main proposed mechanisms describing NP transportation across the BBB,^35^ including opening BBB tight junctions (TJs), endothelial cell transcytosis, and endothelial cell endocytosis. In the latter, the NP content is released into the cell cytoplasm, followed by exocytosis on the abluminal side of the endothelium. PLGA has been approved by US FDA to use in drug delivery systems as a safe, effective, and biodegradable carrier,^25^ with no brain toxicity at a dose of 18 mg/kg,^50^ which is equivalent with our administered PLGA dose (17.74 mg/kg) in rats, excluding the possibility of TJs opening due to NP local toxicity. In view of the fact that SCU-PLGA NPs are only modified by PEG without any ligands and antibodies, the ability to cross intact BBB through the endocytosis is very limited. However, the compromised paracellular barrier caused by TJ protein disassembly in the BBB, as observed in a range of pathological conditions, such as MCAO injury, can facilitate NP accumulation in the brain ischemic lesions.^51^ The reported enlarged TJ gaps (0.2–1.2 μm) with their extended opening up 48 h following stroke^52^ could justify the high accumulation of our small SCU-PLGA NPs (180–200 nm), as reported with other types of NPs.^53,54^ Nevertheless, the exact mechanism of SCU-PLGA NPs crossing the BBB in a stroke model remains to be elucidated.

As a summary, our formulation can protect SCU from the complex biological environment, thereby extending the half-life. To the best of our knowledge, this is the first report of successful fabrication of SCU-PLGA NPs with high extended blood circulation and superior activity against cerebral ischemia. These SCU-NPs could be used in combination with the conventional thrombolytic therapy to improve stroke management in patients.

## 5 Conclusions

Encapsulating SCU into PLGA NPs enhanced its stability, prolonged the SCU residency in the blood circulation, and increased its level in the blood and ischemic brain following intravenous administration. More importantly, a range of histological examinations (TTC, H&E, and TUNEL) and a behavioral study collectively confirmed the superior therapeutic efficacy of SCU-PLGA NPs, over free SCU, in a transient MCAO rat model. This study highlights the great potential of the developed SCU-PLGA NPs as an injectable formulation to treat ischemic cerebrovascular diseases.

## Abbreviations

CCA: common carotid artery
DAPI: 4′,6-diamidino-2-phenylindole
DL: drug loading
ECA: external carotid artery
EDTA-2Na: disodium ethylenediaminetetraacetate
EE: encapsulation efficiency
ESI: electrospray ionization
FITC: fluorescein isothiocyanate
HE: hematoxylin and eosin
I/R: ischemia/reperfusion
ICA: internal carotid artery
LC: liquid chromatography
MCA: middle cerebral artery
MCAO: middle cerebral artery occlusion
NPs: nanoparticles
PDI: polydispersity index
PEG-PLGA: poly(ethylene glycol) methyl ether-*block*-poly(lactide-*co*-glycolide)
PGA: poly(glycolic acid)
PLA: poly(lactic acid)
PLGA: 50:50 dl-lactide/glycolide copolymer
PVA: poly(vinyl alcohol)
SCU: scutellarin
SD: standard deviation
SD rat: Sprague-Dawley rat
SIR: selected ion recording
TCM: traditional Chinese medicine
TEM: transmission electron microscopy
TTC: 2,3,5-triphenyltetrazolium chloride
TUNEL: terminal deoxynucleotidyl transferase-mediated dUTP nick-end labeling
UPLC–MS: ultraperformance liquid chromatography–mass spectrometry.
